# The Value of Transvaginal Neurosonography in the Detection of Fetal Ganglionic Eminence Abnormalities

**DOI:** 10.3390/life16081362

**Published:** 2026-08-19

**Authors:** Krzysztof Berbeka, Aleksy Świetlicki, Katarzyna Stefańska, Dorota Sys, Dagmara Filipecka-Tyczka, Evelina Bertelli, Manuela Tavares De Sousa, Igor Hawryluk, Magdalena Rudzińska, Sambor Sawicki, Miriam Illa

**Affiliations:** 1 First Department of Obstetrics and Gynecology, Centre of Postgraduate Medical Education, ul. Żelazna 90, 01-004 Warsaw, Poland; berbekakrzysztof@gmail.com (K.B.); d.filipecka@gmail.com (D.F.-T.); 2 Żelazna Medical Center, St. Sophia’s Specialist Hospital, ul. Żelazna 90, 01-004 Warsaw, Poland; hawryluk.igor@gmail.com (I.H.); m.rudzinska@szpitalzelazna.pl (M.R.); 3 Department of Reproduction, Chair of Fetomaternal Medicine, Poznan University of Medical Sciences, ul. Polna 33, 60-535 Poznan, Poland; aleksy.swietlicki@gmail.com; 4 Doctoral School, Poznan University of Medical Sciences, ul. Bukowska 70, 60-812 Poznan, Poland; 5 Department of Gynecology and Obstetrics, Medical University of Gdansk, ul. Smoluchowskiego 17, 80-952 Gdansk, Poland; sambor.sawicki@gumed.edu.pl; 6 Department of Translational Immunology and Experimental Intensive Care, Centre of Postgraduate Medical Education, 01-813 Warsaw, Poland; dorota.sys@cmkp.edu.pl; 7 Obstetrics and Gynaecology Department, IRCCS San Raffaele Scientific Institute, Vita-Salute San Raffaele University, Via Olgettina 58, 20132 Milan, Italy; bertellievelina@gmail.com; 8 Department of Fetal Medicine and Obstetrics, University Medical Center Hamburg-Eppendorf, Martinistraße 52, Building O10, 20246 Hamburg, Germany; m.tavares-de-sousa@uke.de; 9 BCNatal, Fetal Medicine Research Center (Hospital Clinic and Hospital Sant Joan de Déu), Universitat de Barcelona, Carrer de Mejía Lequerica, 39, 08028 Barcelona, Spain; miriam.illa@medicinafetalbarcelona.org; 10 Institute De Recerca Sant Joan De Déu (IRSJD), Fundació Sant Joan De Déu, Santa Rosa, 39-57, 08950 Esplugues de Llobregat, Spain; 11 Spanish Network in Maternal, Neonatal, Child and Developmental Health Research (RICORS-SAMID, RD24/0013/0004), Instituto de Salud Carlos III, 28040 Madrid, Spain

**Keywords:** neurosonography, fetal brain, ganglionic eminence, ultrasound, cortical development

## Abstract

Introduction: The fetal ganglionic eminence (GE) is a transient brain structure involved in interneuron development; its abnormalities may be associated with malformations of cortical development (MCD). This systematic review evaluated the feasibility and clinical value of transvaginal neurosonography for fetal GE assessment. Material and Methods: PubMed, Scopus, and Web of Science were searched from January 2015 to June 2026. Human in vivo ultrasound studies of the fetal GE were included. Results: Thirteen studies comprising 890 fetuses were included. GE visualization ranged from 71% to 100%, with highest rates using dedicated three-dimensional transvaginal neurosonography at 9–22 weeks. Measurement reproducibility was good to excellent. GE cavitation and enlargement were associated with MCD in selected high-risk cohorts, but reported frequencies varied substantially. Conclusions: Transvaginal neurosonography permits feasible and reproducible visualization of the fetal GE in experienced hands. Small studies, heterogeneous protocols, and limited long-term outcome data preclude routine GE assessment at present.

## 1. Introduction

The ganglionic eminence (GE) is a transient structure in early fetal brain development that plays a key role in neuronal migration and cortical organization [1,2,3,4,5]. Its prenatal identification is increasingly reported due to advances in neurosonography, yet its normal and abnormal sonographic appearances remain incompletely defined. Located inferolateral to the frontal horns of the lateral ventricles, the GE contributes to the development of GABAergic interneurons and other neural cell lineages. It also contributes to a population of thalamic neurons and is responsible for forming most of the basal ganglia. Moreover, the GE represents an intermediate target for thalamocortical and corticothalamic axons and is a source of oligodendrocyte precursors [1,2,3,4,5]. The GE development involves three distinct regions: Medial Ganglionic Eminence (MGE), Lateral Ganglionic Eminence (LGE) and Caudal Ganglionic Eminence (CGE) [6,7]. Their progenitor cells are located primarily in the MGE and CGE, with a smaller contribution from the LGE and the preoptic region [8,9,10].

Studies using three-dimensional brain organoids have provided a broad understanding of the models and mechanisms of neuronal migration. These investigations have shown that human cortical interneurons migrate in a saltatory manner, involving the extension and remodeling of branched neural processes [11,12,13]. Over the years, evidence has accumulated suggesting that GABAergic interneurons have important roles in many cortical functions. Consistent with this role, malfunction of inhibitory interneurons has been associated with therapy-resistant epilepsy, severe neurodevelopmental delay, schizophrenia, anxiety disorders and autism [14,15,16].

Alterations in GE morphology or echogenicity have been correlated with various neuronal migration disorders, highlighting its predictive value in identifying central nervous system (CNS) malformations such as lissencephaly, schizencephaly, and heterotopia [17,18,19]. Also such alterations as reduced gyration and opercularization as well as callosal agenesis, vermian and cerebellar anomalies, ventriculomegaly, enlarged subarachnoid spaces, molar tooth sign and microcephaly have been detected [17,18,19,20]. In most studies, GE abnormalities have been categorized into two main groups: those associated with cavitation and those associated with enlargement [21].

The ganglionic eminence is a transient telencephalic compartment that is available for sonographic assessment from approximately 7–8 weeks of gestation [22,23]. It reaches maximal size between 14 and 20 weeks and then undergoes physiological involution between 20 and 28 weeks, with only limited visibility by 28–32 weeks [2,4,24]. Transvaginal ultrasound is preferred for its assessment because it provides higher spatial resolution than the transabdominal approach, particularly during the first and early second trimester [2]. A dedicated acquisition using the coronal plane is optimal for GE evaluation, while the parasagittal plane can add supplementary information [4]. Three-dimensional ultrasound with volume rendering (e.g., HDlive) supports multiplanar review and improves morphological assessment compared with two-dimensional imaging alone [21,22,23,24,25]. Dedicated transvaginal neurosonography was typically performed using high-frequency probes (5–9 MHz or higher). Optimal settings included high-resolution presets, low persistence, and harmonic imaging when available. The coronal and parasagittal planes were used for GE evaluation.

**Figure 1:**
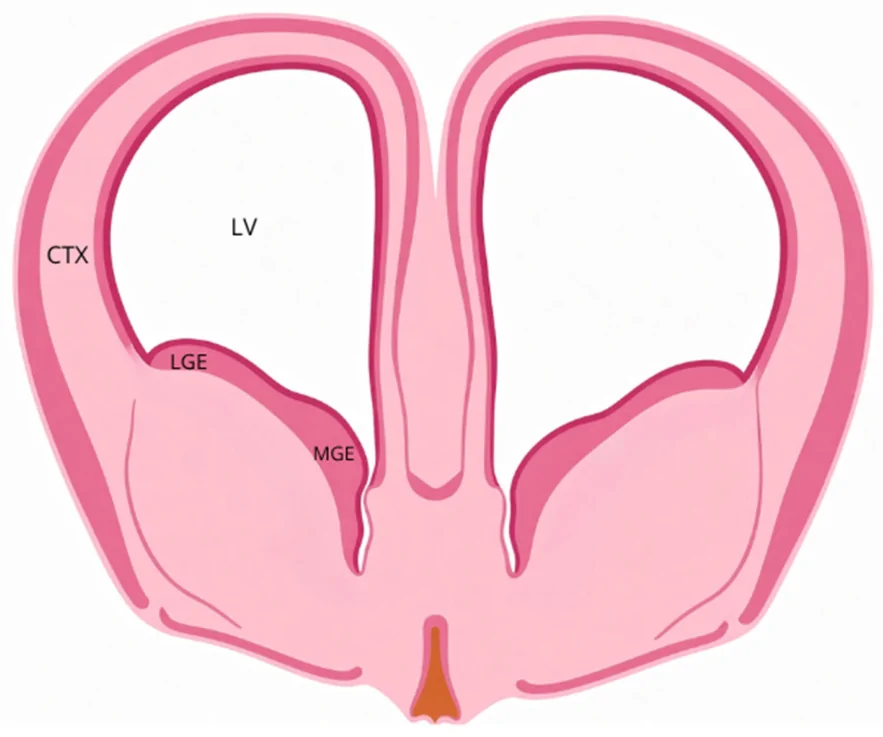
Schematic coronal illustration of the normal fetal ganglionic eminence (GE). The lateral ganglionic eminence (LGE) and medial ganglionic eminence (MGE) are shown along the inferolateral walls of the lateral ventricles (LV), beneath the developing cerebral cortex (CTX). This illustration is intended to facilitate anatomical orientation during fetal neurosonographic assessment.

**Figure 2:**
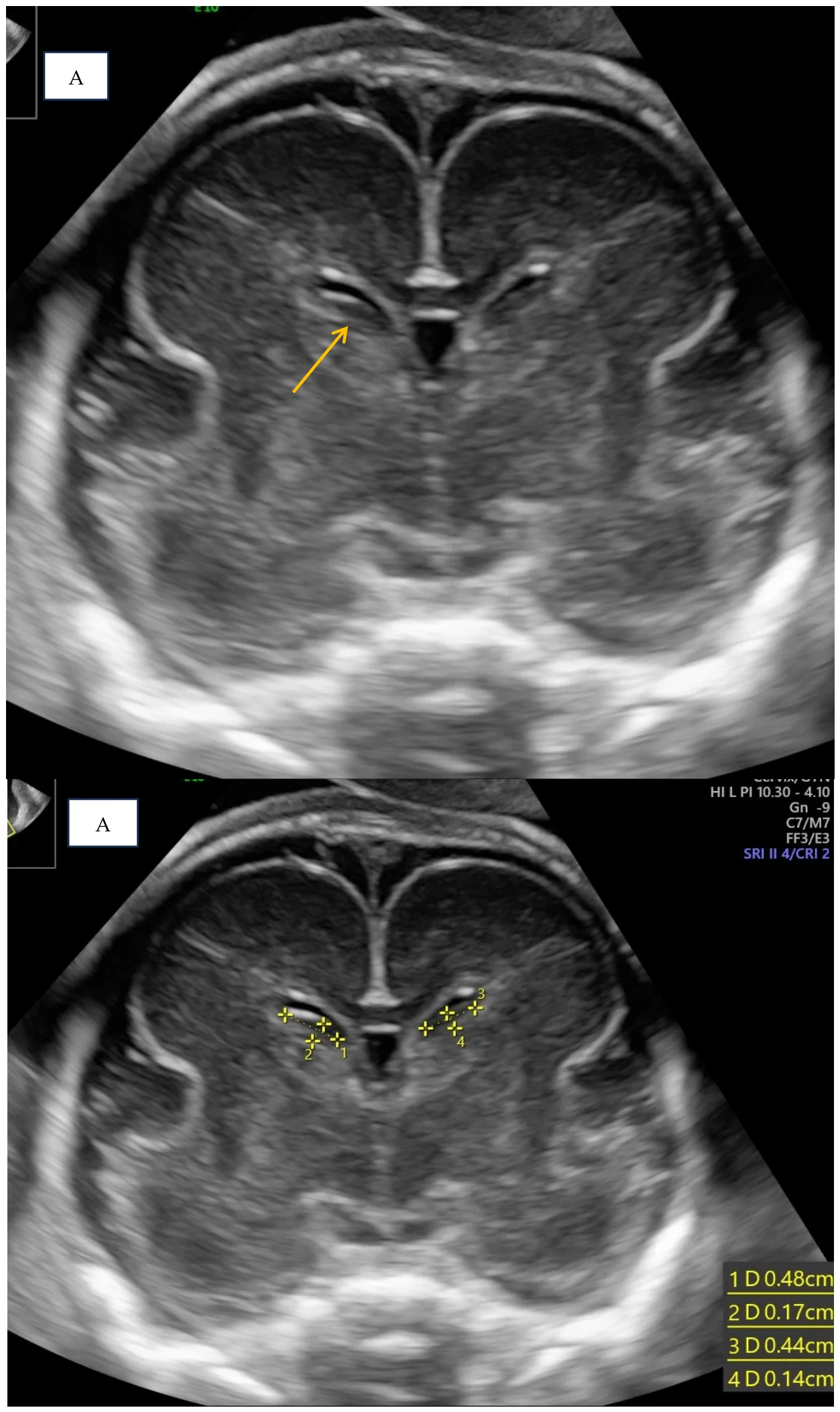

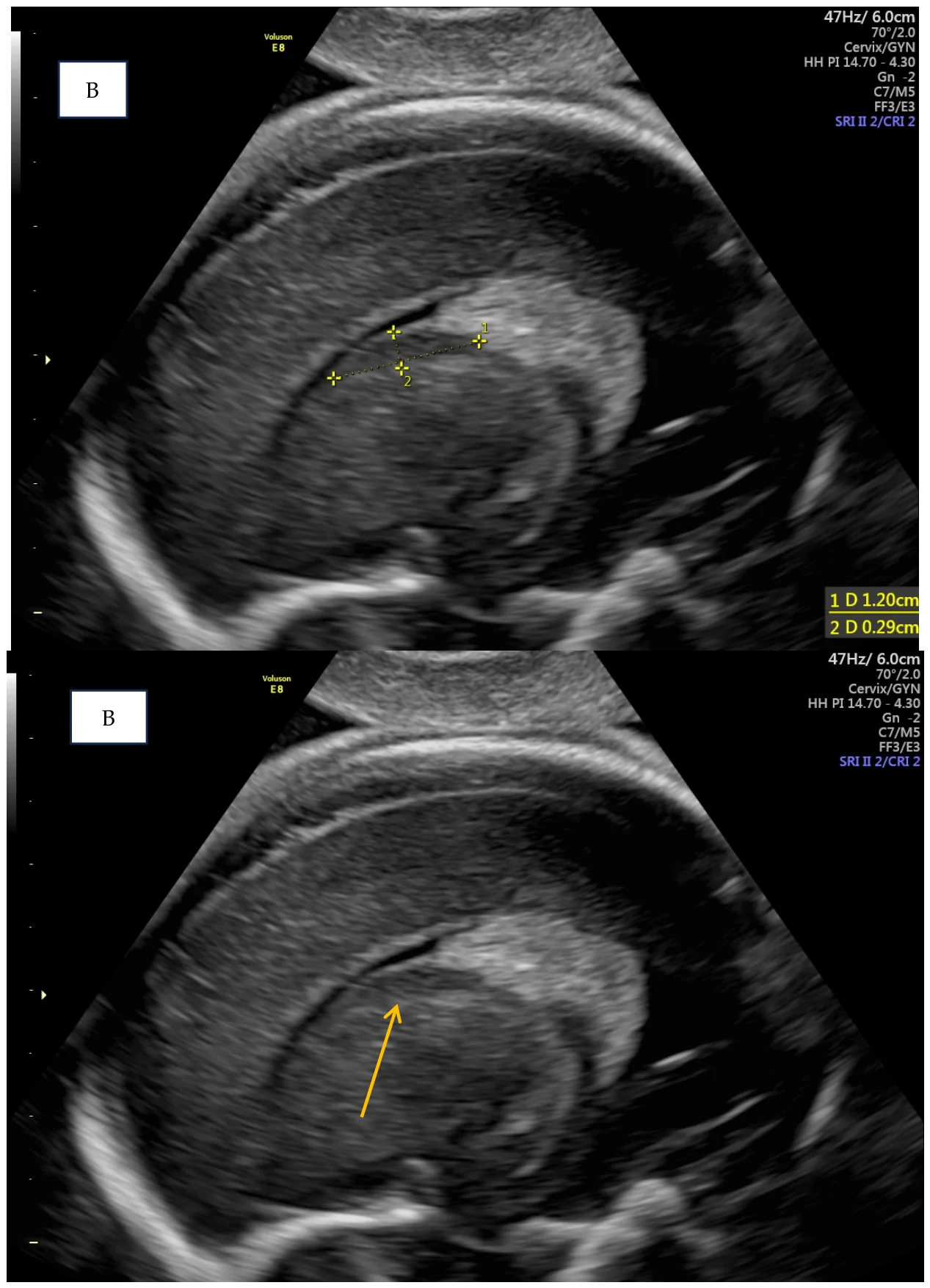
Standard ultrasound planes for the assessment of the fetal ganglionic eminence (GE) using transvaginal neurosonography at 20 weeks of gestation. (**A**) Coronal view showing the GE (arrow) located inferolateral to the frontal horns of the lateral ventricles. (**B**) Parasagittal view demonstrating the normal appearance of the GE (arrow) anterior and inferior to the choroid plexus within the caudothalamic groove. This plane is particularly useful for detailed morphological evaluation and measurements of the GE.

A schematic coronal illustration of the normal GE is presented in Figure 1. A normal visualization of GE is presented in Figure 2. On a properly obtained view, the GE lies at the floor of the lateral ventricle, within the caudothalamic groove, between the head of the caudate nucleus and the thalamus. It is located anterior and medial to the choroid plexus, which serves as the key anatomical landmark for distinguishing GE abnormalities from nearby cystic lesions [2,4]. In normal development, the GE appears as a smooth, slightly prominent ridge with homogeneous echotexture and isoechoic to mildly hyperechoic signal relative to adjacent brain tissue [21]. Cystic spaces are not expected in the GE in normal development [4,6]. From approximately 11 weeks onward, the GE can be measured in longitudinal and transverse dimensions, with better reproducibility reported for the longitudinal diameter [4].

GE assessment should be performed using a targeted transvaginal neurosonographic examination, preferably with acquisition of a three-dimensional volume that permits standardized multiplanar review. The coronal plane is useful for recognizing the GE as a smooth structure inferolateral to the frontal horns of the lateral ventricles. For measurement, a parasagittal plane through the caudothalamic groove should be obtained, with the GE identified anterior and inferior to the choroid plexus. The longitudinal diameter is measured along the longest visible axis of the GE, whereas the transverse diameter is measured perpendicular to this axis at its greatest width. Measurements should be obtained on a magnified, frozen image with clear delineation of the GE margins and recorded together with gestational age and imaging plane. When an abnormal GE appearance is suspected, the examination should include a systematic assessment of ventricular morphology, cortical maturation and gyration, midline structures including the corpus callosum, and the posterior fossa. Particular attention should be paid to additional features suggestive of malformations of cortical development, such as delayed gyration, abnormal opercularization, irregular cortical contour, or heterotopia when detectable. Three-dimensional volume acquisition may facilitate multiplanar review and documentation in expert examinations [17,18,19].

The ganglionic eminence is a critical subpallial source of cortical inhibitory interneurons and therefore a key upstream determinant of cortical circuit development. The medial ganglionic eminence (MGE) generates the majority of cortical GABAergic interneurons, including parvalbumin- and somatostatin-expressing subtypes, whereas the lateral ganglionic eminence (LGE) produces striatal projection neurons and interneuron lineages that can contribute to the cerebral cortex [6]. After specification within the ventral telencephalon, these interneurons migrate tangentially over millimeter distances to reach their cortical destinations. Their trajectories and eventual subtype integration are controlled by coordinated molecular guidance and transcriptional programs, including region- and time-specific regulatory networks and guidance cues (e.g., DLX transcriptional programs, ErbB4, CXCR4, and semaphorin pathways) [7]. Disruption of GE integrity during the window when interneurons are generated and instructed to migrate is therefore expected to impair interneuron production, migration, and/or specification. Because GABAergic interneurons shape cortical excitability and support the formation of balanced inhibitory circuits, loss of correct interneuron number, subtype identity, or positioning can translate into a spectrum of malformations of cortical development [8]. Histopathological processes underlying sonographic GE abnormalities, such as necrosis or hemorrhage within GE tissue (cavitation), dysregulated cellular proliferation (enlargement), or abnormal persistence of the germinal zone (failed involution/persistence) map directly onto disrupted interneuron ontogenesis. This mechanistic framework also explains the shared pathological thread across lissencephaly, periventricular heterotopia, polymicrogyria, and schizencephaly: impaired neuronal migration and disrupted cortical organization. Consistent with a more recently proposed enrichment of mitochondria-related genetic dysfunction in GE cysts, impaired cellular energy homeostasis may further compromise progenitor survival and energy-dependent migration, adding a novel layer to how GE pathology translates into cortical phenotype [26]. A further consequence is that prenatal neurosonography targets a biologically meaningful period, because the GE is most active as a germinal zone during mid-gestation, aligning imaging feasibility with the timing of the mechanistic steps that determine downstream cortical outcomes.

**Figure 3:**
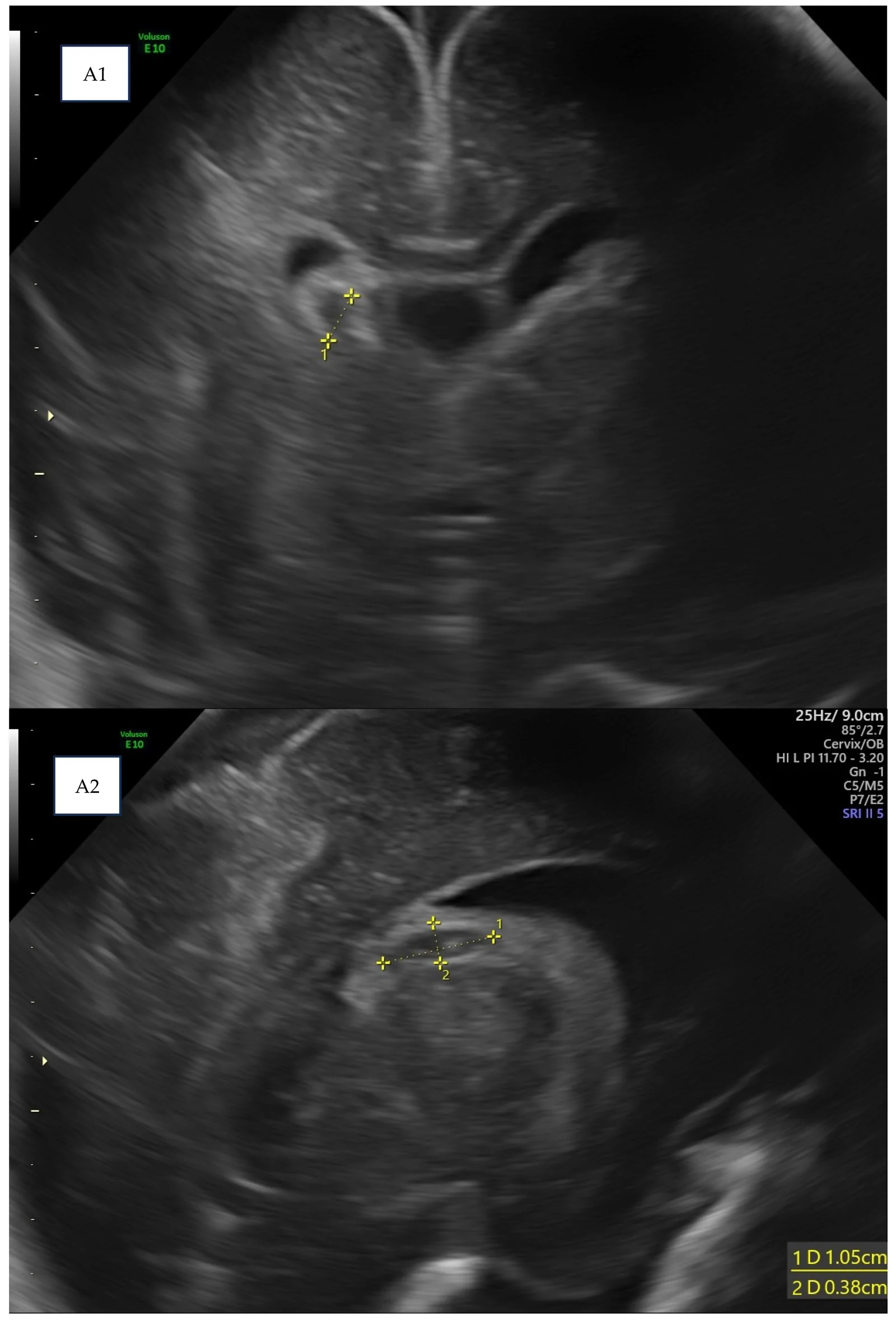

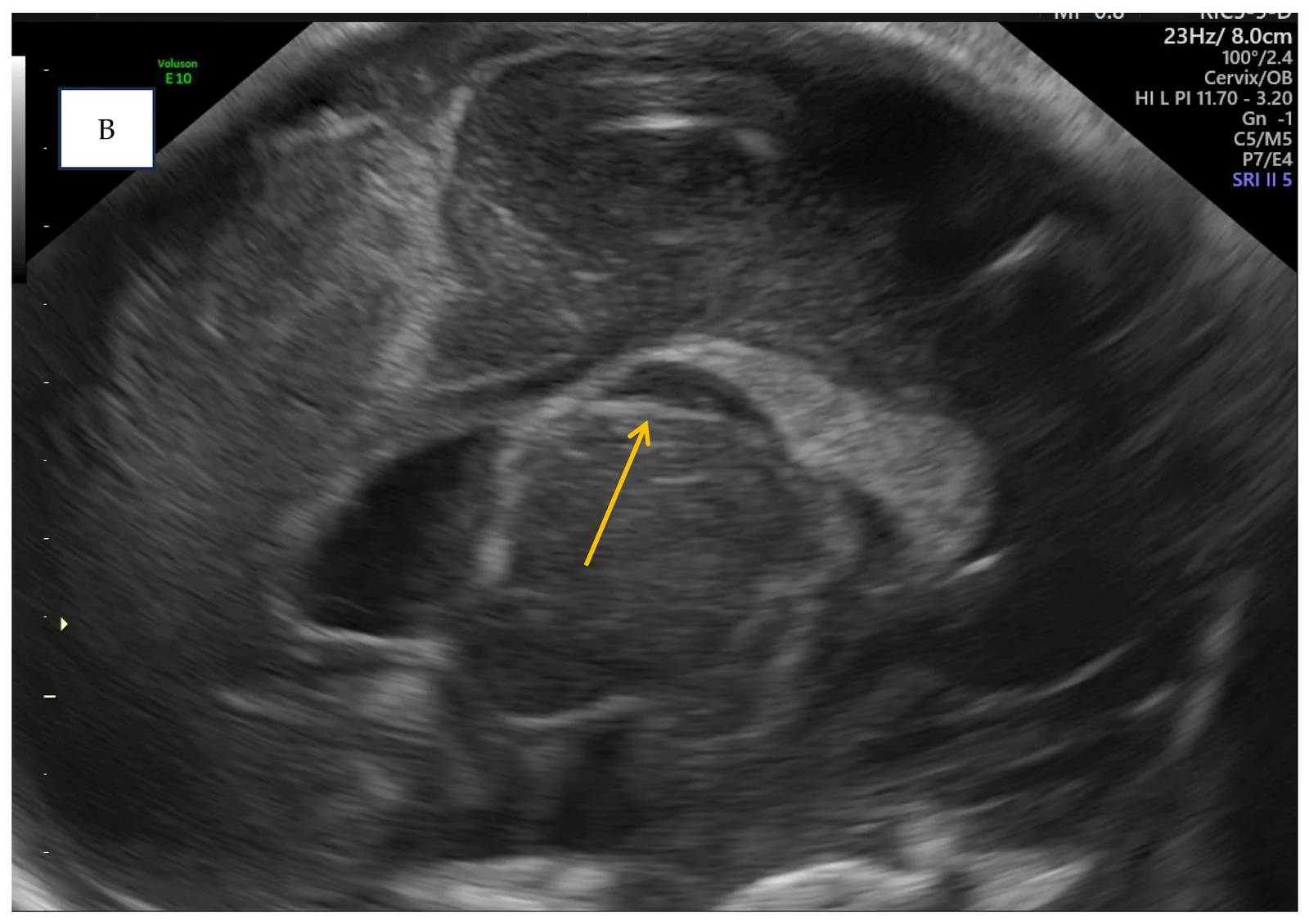
Representative transvaginal neurosonographic images of ganglionic eminence (GE) abnormalities at 20 weeks of gestation. (**A**) GE cavitation demonstrated in the coronal plane (**A1**) and parasagittal plane (**A2**). Note the characteristic hypoechoic cystic areas located anterior to the choroid plexus (indicator). (**B**) GE enlargement shown in the parasagittal plane with marked bulging of the GE into the lateral ventricle (arrow). GE enlargement is characterized by increased prominence and convex bulging of the GE into the lateral ventricle, with loss of its normal smooth ridge-like contour.

When GE pathology is suspected, two core abnormal patterns are typically considered: cavitation and enlargement. Cavitation refers to one or more anechoic spaces within GE tissue, and the most practical discriminator is that GE cavitation lies anterior to the choroid plexus, helping to distinguish it from choroid plexus cysts that are more posteriorly located [27,28]. Enlargement refers to a disproportionately increased GE size with greater prominence or bulging into the ventricular lumen, and persistence beyond the expected involution period should be interpreted with gestational age in mind. Cavitation is not considered a normal finding at any gestational age [4]. Typical examples of GE cavitation and GE enlargement on transvaginal ultrasound are shown in Figure 3.

**Figure 4:**
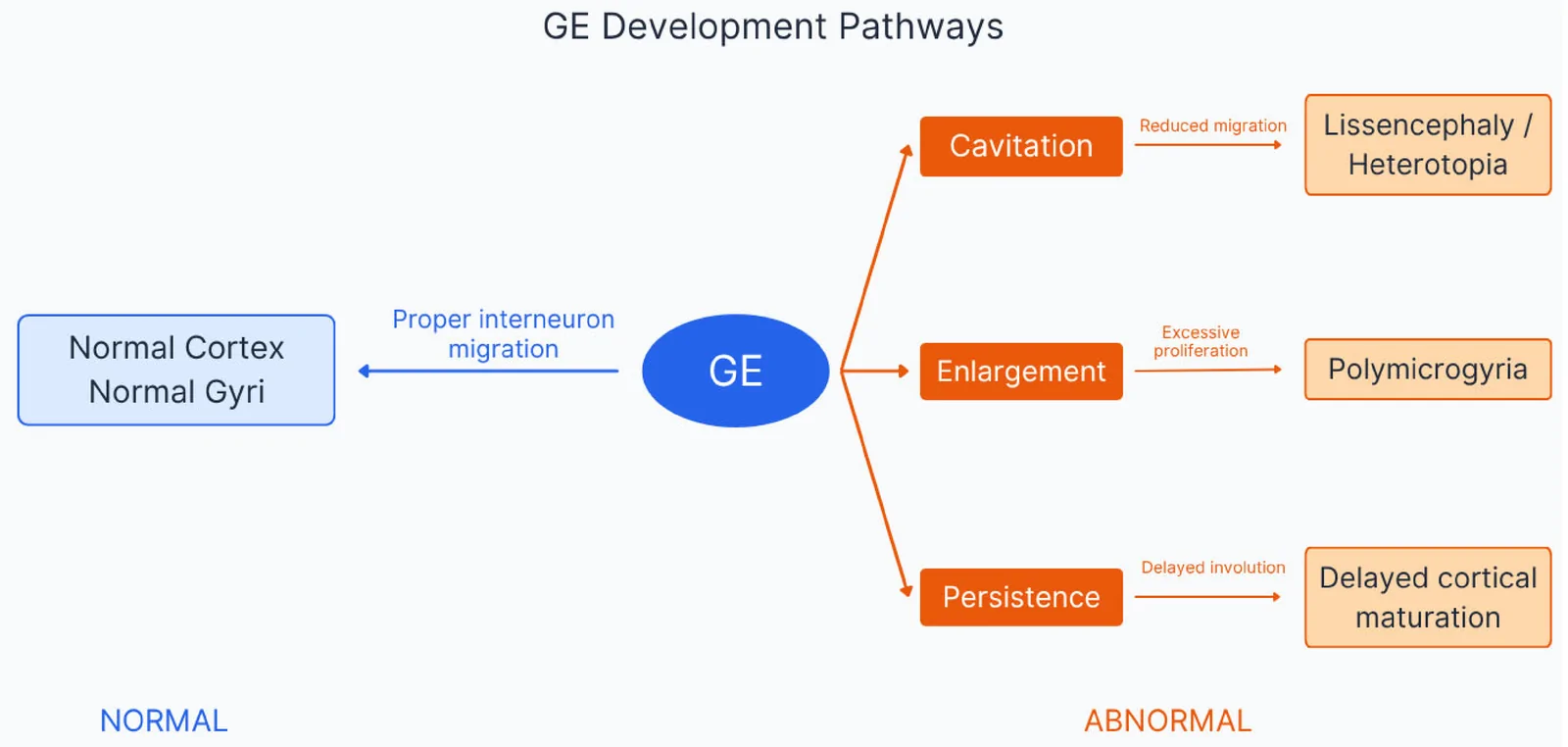
Schematic representation of normal and abnormal ganglionic eminence (GE) development and its relationship with malformations of cortical development (MCD). In normal development (**left panel**), the GE acts as the main source of GABAergic interneurons, which migrate tangentially to the cerebral cortex, resulting in proper cortical organization and gyration. In abnormal conditions (**right panel**), GE cavitation leads to a reduced progenitor cell pool and impaired migration (commonly associated with lissencephaly and heterotopia), while GE enlargement is linked to excessive progenitor proliferation and disorganized migration (commonly associated with polymicrogyria). GE persistence may result in delayed cortical maturation and sulcation defects.

Figure 4 schematically illustrates the normal development of the ganglionic eminence and the proposed pathophysiological mechanisms linking different types of GE abnormalities to specific cortical malformations through disruption of interneuron generation and migration.

Despite growing interest in prenatal GE imaging, there is currently no clear consensus on the spectrum of normal versus abnormal sonographic findings, their reproducibility across different operators and centers, or their clinical significance for prenatal counseling and management. This systematic review aims to synthesize the available evidence on prenatal ultrasound assessment of the ganglionic eminence, including its visualization, reproducibility, and spectrum of abnormalities, and to evaluate its potential role in the early identification of central nervous system malformations.

The clinical impact of reliable prenatal assessment of the ganglionic eminence is potentially significant. Because GE abnormalities are rarely isolated and are frequently associated with malformations of cortical development, their early detection may facilitate timely referral for fetal MRI, genetic testing (with a diagnostic yield of 40–71% in the reviewed studies), and multidisciplinary counseling. Furthermore, identification of GE pathology can influence decisions regarding delivery in a tertiary center equipped for neonatal neurology support. Despite these important implications, the feasibility, reproducibility, and diagnostic value of ultrasound in GE evaluation have not been systematically synthesized until now.

## 2. Methods and Materials

This systematic review was conducted and reported in accordance with the PRISMA 2020 statement. The protocol was prospectively registered in PROSPERO (CRD420251178644). A MOOSE checklist is provided as Supplementary Material.

### 2.1. Eligibility Criteria

Studies were eligible if they reported in vivo ultrasound imaging (any modality) of the fetal ganglionic eminence (GE) in human pregnancies. Eligible studies included retrospective, prospective, cross-sectional, or case report/case series designs, were published in English between January 2015 and June 2026, and provided outcome data on GE visualization, measurement, abnormalities, or associated malformations. No restrictions were applied on gestational age, sample size, or the presence of a comparator. Studies were excluded if they were conducted on animal models, used postmortem or ex vivo imaging, relied on MRI as the sole imaging modality, or were reviews, editorials, conference abstracts without full data, or letters without original data.

### 2.2. Information Sources and Search Strategy

Electronic databases searched: PubMed/MEDLINE, Scopus, and Web of Science. No additional sources (grey literature, trial registries, hand-searching) were used due to the specificity of the topic and time constraints. The search was conducted from January 2015 to February 2025. An updated search was conducted in June 2026 to capture publications from March 2025 to June 2026, covering PubMed, Scopus, and Web of Science, using identical search terms as the original search. The following keywords were combined with Boolean operators: “fetal ganglionic eminence” OR “ganglionic eminence” OR “ganglionic eminence anomaly” OR “ganglionic eminence cavitation” OR “ganglionic eminence imaging” OR “ganglionic eminence abnormalities” OR “ganglionic eminence cysts” AND (“ultrasound” OR “ultrasonography” OR “neurosonography” OR “transvaginal ultrasound” OR “3D ultrasound” OR “4D ultrasound”). Full search strings for each database are presented in Table S1.

### 2.3. Selection Process

**Figure 5:**
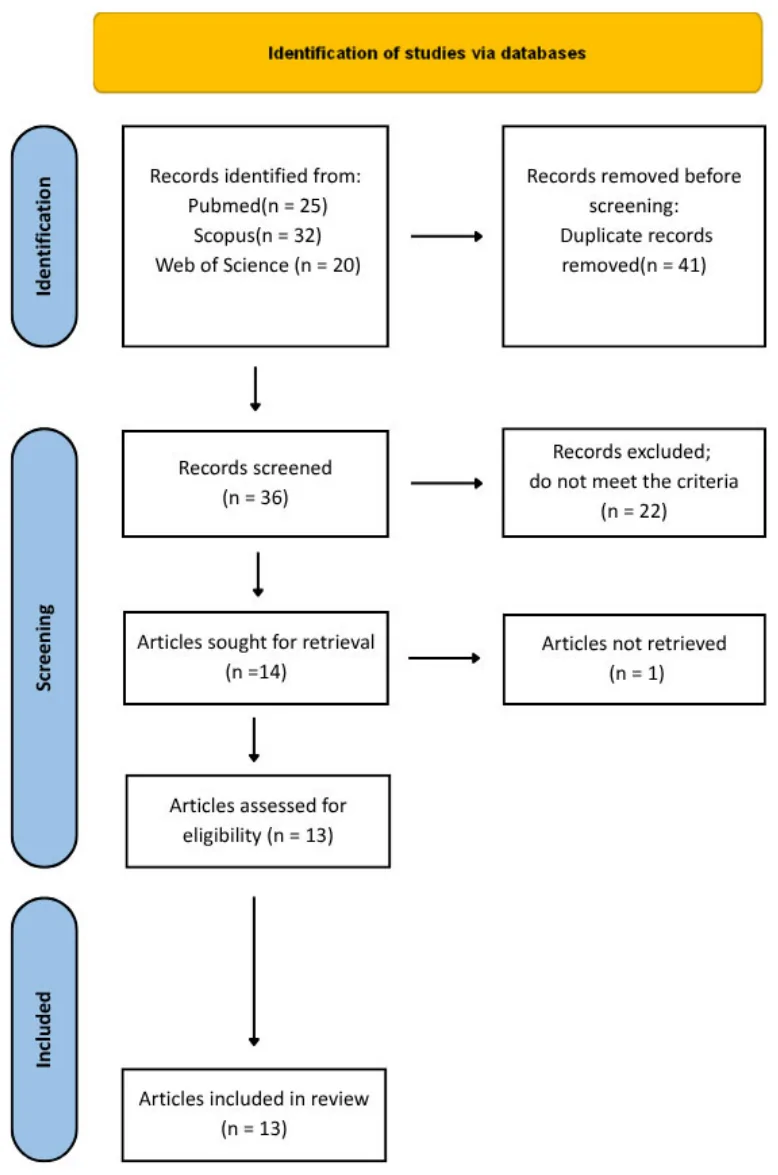
PRISMA flowchart.

Titles and abstracts were screened independently by two reviewers (KB and AS). Disagreements were resolved by discussion or consultation with a third reviewer (DF). Full texts of potentially eligible records were retrieved and assessed independently by the same two reviewers. Reasons for exclusion at full-text stage were recorded. The study selection process is presented in the PRISMA flow diagram (Figure 5).

### 2.4. Data Collection Process

Data were extracted independently by two reviewers (KB and AS) using a standardized form. Disagreements were resolved by discussion or third reviewer adjudication (DF). Extracted items included: study design, sample size, gestational age, imaging protocol, visualization rate, reproducibility measures (e.g., ICC), types and prevalence of GE abnormalities, association with MCD/CNS anomalies, gestational age at first visualization, distinction from choroid plexus cysts, and clinical implications (referral to MRI, genetic testing, tertiary care).

### 2.5. Data Items

Data were extracted from each included study using a standardized form. The following variables were collected: first author and year of publication, study design, period of patient recruitment, number of fetuses examined, gestational age at ultrasound examination, type of ultrasound protocol (transvaginal/general, 2D/3D, dedicated neurosonography or not), visualization rate of the ganglionic eminence, reproducibility measures (e.g., intra- and interobserver intraclass correlation coefficients), prevalence and types of GE abnormalities (cavitation, enlargement, persistence), number of cases with each type of abnormality, association of GE abnormalities with malformations of cortical development or other central nervous system anomalies, gestational age at first visualization of GE and clinical implications (referral rates for fetal MRI, genetic testing using chromosomal microarray or exome sequencing, and delivery in a tertiary care center). No imputation was performed for missing data.

### 2.6. Study Risk of Bias Assessment

Risk of bias was assessed using the QUADAS-2 tool for cohort and case–control (*n* = 9) and the Joanna Briggs Institute (JBI) critical appraisal checklist for case reports and case series (*n* = 4). QUADAS-2 domains included patient selection, index test, reference standard, and flow and timing. JBI domains evaluated clarity of reporting, patient characteristics, diagnostic procedures, and outcome assessment. Assessments were performed independently by two reviewers; disagreements were resolved by consensus. Plots are generated by robvis tool [29].

### 2.7. Effect Measures

No formal effect measures were calculated due to the heterogeneity of study designs, populations, and outcomes.

### 2.8. Synthesis Methods

Narrative synthesis was performed due to clinical and methodological heterogeneity (different designs, gestational age ranges, imaging protocols, and outcome definitions). Meta-analysis was not conducted. Synthesis was structured by key outcomes: feasibility of visualization, reproducibility, prevalence and types of abnormalities, association with MCD/CNS anomalies, and clinical implications. No subgroup or sensitivity analyses were performed.

## 3. Results

### 3.1. Study Selection

The initial database search (January 2015 to February 2025) identified 48 records, of which 25 were duplicates. After de-duplication, 23 records remained for title and abstract screening. Eleven full-text articles were assessed for eligibility; 12 records were excluded after full-text review for not meeting the inclusion criteria, yielding 11 studies for inclusion.

An updated search (March 2025 to June 2026) across the same three databases identified 27 additional records (Scopus *n* = 12, Web of Science *n* = 7, PubMed *n* = 8), of which 14 were duplicates. After de-duplication, 13 unique records remained. Ten records were excluded at the title and abstract screening stage (7 used MRI as the sole imaging modality, 1 was a post-mortem study, 1 was unrelated to the topic, and 1 was a single case report with insufficient data for synthesis). Three full-text articles were assessed for eligibility; one was excluded because ganglionic eminence assessment was performed using MRI rather than ultrasound. Two studies met the inclusion criteria and were added to the review.

Ultimately, 13 studies (total 890 fetuses) were included in the updated review (Figure 5).

### 3.2. Study Characteristics

The included studies were published between 2015 and 2026 and involved a total of 890 fetuses (range 2–402 per study). Study designs comprised prospective cohort/multicenter (*n* = 4) [18,21,22,24], retrospective cohort/multicenter (*n* = 5) [17,19,23,25,30], and case reports/series (*n* = 4) [26,27,28,31]. Gestational age at examination ranged from 7–10 weeks to 35 weeks, with most studies focusing on 11–22 weeks. Imaging modalities included dedicated transvaginal 3D neurosonography (*n* = 6) [21,22,23,24,25,26], conventional transvaginal ultrasound (*n* = 2) [27,28], combined US/MRI (*n* = 3) [17,18,19], and multimodal neurosonography (*n* = 1) [30]. One study used an unspecified protocol [31]. Populations were unselected/normal in 5 studies and high-risk/MCD-suspected in 8. The characteristics of the included studies are summarized in Table 1.

### 3.3. Risk of Bias in Studies

Risk of bias was assessed using QUADAS-2 for diagnostic accuracy studies (*n* = 9) and JBI checklist for case reports/series (*n* = 4). Prospective studies with dedicated neurosonography protocols had a low risk of bias in patient selection, index test, and timing. Retrospective and case-report studies showed high risk in patient selection and applicability due to small sample sizes and retrospective nature. No study had high risk in reference standard (MRI or postnatal confirmation when available).

Of the two studies added in the updated search, Chen et al. (2025) [30] was assessed using QUADAS-2 and rated as having high risk of bias, mainly due to incomplete genetic evaluation (complete CMA and exome sequencing available in only 42% of fetuses) and substantial loss to follow-up. Birnbaum et al. (2026) [26] was assessed using the JBI checklist for case series and rated as having moderate risk of bias, primarily due to non-consecutive patient recruitment and referral bias, both explicitly acknowledged by the authors.

All assessed case reports and case series demonstrated generally high reporting quality. Patient characteristics, clinical presentation, diagnostic procedures, and follow-up outcomes were clearly described in all included publications. Diagnostic evaluation was consistently based on prenatal ultrasound, frequently complemented by fetal MRI. Potential harms were mainly related to diagnostic misclassification, particularly confusion with choroid plexus cysts. Overall, the studies provided clear clinical insights and were therefore retained for inclusion in the qualitative synthesis.

Summary of risk of bias is presented in Figures S1 and S2.

### 3.4. Feasibility of GE Visualization

Across studies, GE visualization by transvaginal ultrasound was feasible, with reported visualization rates ranging from 71% to 100%, and higher rates in studies using dedicated 3D neurosonography protocols (Table 2). Feasibility varied by GA and protocol. In the earliest gestation pilot, the embryonic GE was visualized in all 4 fetuses (100%) at 7–10 weeks using 3D/4D transvaginal ultrasound [23]. At 9–13 weeks, GE was visualized in 15/18 fetuses (83%) using 3D transvaginal ultrasound with HDlive rendering [22]. In the largest prospective first-trimester series (11+3 to 13+6 weeks; *n* = 402), GE was identified in 285/402 fetuses (71%), and at least one dimension could be measured in 229/402 fetuses (57%) [24]. In the early second trimester, GE was visualized in all 84 fetuses (100%) at 14–18 weeks using a 3D transvaginal parasagittal approach [25]. At mid-gestation (19–22 weeks), GE was identified in 144/160 normal fetuses (90%) [21]. Overall, these data suggest that dedicated transvaginal 3D neurosonography achieves consistently high visualization rates, whereas general ultrasound protocols did not consistently report GE-specific visualization and may underperform for this target structure.

Reproducibility was assessed in two prospective studies. Contro et al. reported intraobserver ICC of 0.90 (95% CI 0.83–0.93) for the longitudinal diameter and interobserver ICC of 0.90 (95% CI 0.86–0.92) for the same dimension; the transverse diameter showed lower reproducibility (intraobserver ICC 0.80; interobserver ICC 0.64) [21]. Altmann et al. reported almost perfect intraobserver agreement and interobserver ICC values ranging from 0.64 to 0.90 depending on the measured dimension [24].

The feasibility pattern across gestation is summarized below to emphasize the practical diagnostic window reported in the included studies (Table 3). GE can be visualized across a wide GA range, but the most reliable and reproducible ultrasound assessment is reported from 14 weeks onward, particularly with dedicated transvaginal 3D neurosonography (optimal window 14–22 weeks). Of the two studies added in the updated search, Birnbaum et al. confirmed successful GE cyst visualization in all 25 referred cases (100%) using dedicated transvaginal 3D neurosonography between 11+3 and 22+4 weeks, consistent with the feasibility data from the original search [26]. Chen et al. did not report a GE-specific visualization rate, as GE assessment (sign I) was evaluated within a broader neurosonographic protocol covering 10 MCD-related signs [30].

### 3.5. Diagnostic Performance: Ultrasound Versus MRI

In the only included study providing a direct within-cohort comparison of modalities, Righini et al. reported GE anomalies in all 8 fetuses on fetal MRI at 19–29 weeks, while none were detected on general (non-dedicated) ultrasound [17]. In case-based studies using dedicated transvaginal neurosonography, GE cavitation was detected on ultrasound in three fetuses at 15–20 weeks [28]. Prefumo et al. reported detection of GE cavitation on ultrasound in two fetuses (16 and 20 weeks), with findings described as comparable to MRI [31]. Paladini et al. performed neurosonographic reassessment in seven fetuses referred for large choroid plexus cysts and identified GE cavitation in 4/7 cases [27]. Across cohorts with abnormal GE findings, MRI referral rates ranged from 91% to 100% [18,19].

The two studies added in the updated search provide further data on the complementary role of MRI. Birnbaum et al. performed MRI in selected cases to confirm ultrasound findings and characterize associated anomalies; GE cysts were initially identified or confirmed on dedicated transvaginal neurosonography, with MRI used as a supplementary modality [26]. Chen et al. reported that fetal MRI was performed in 27 of 31 genetically confirmed MCD cases, revealing findings similar to those on neurosonography in 21 fetuses, while providing additional information (abnormal lamination or persistent GE) in the remaining 6, consistent with MRI serving a confirmatory and supplementary rather than a primary diagnostic role [30].

**Figure 6:**
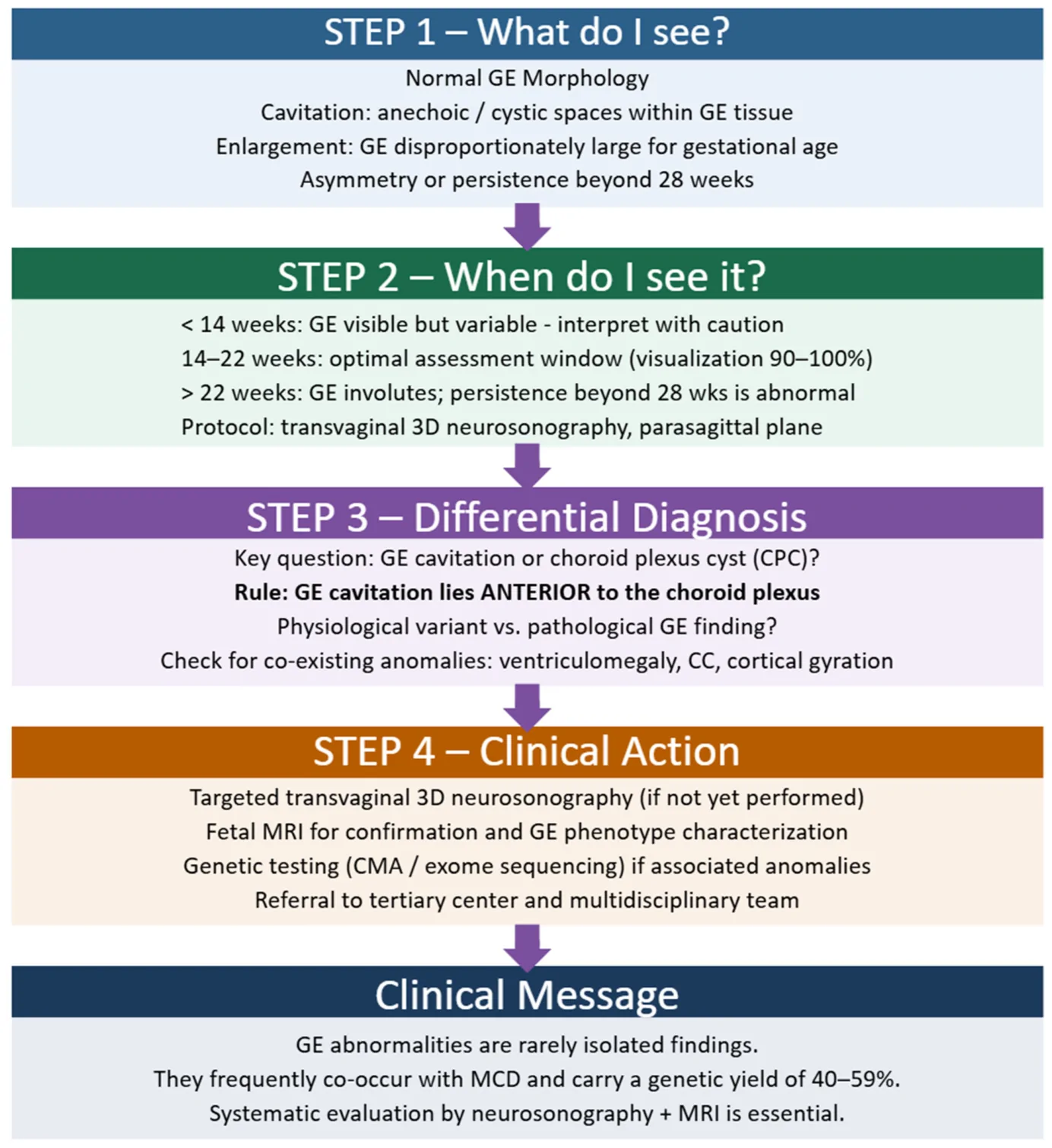
Prenatal GE Assessment: Clinical Decision Framework.

GE Assessment flowchart is presented in Figure 6.

### 3.6. Types of GE Abnormalities

Two main phenotypes of GE abnormality were reported across included studies: cavitation and enlargement. In the largest MRI-based series (*n* = 60; 17–33 weeks), Scarabello et al. reported cavitation in 34/60 cases (57%) and enlargement in 26/60 cases (43%) [19]. Among 50 fetuses with MCD examined by neurosonography, Contro et al. reported GE enlargement in 12/50 cases (24%) and GE cavitation in 4/50 cases (8%) [21]. Persistence of the GE was reported in 32% of one series [18]. Bilateral cavitations were associated with more severe cortical phenotypes [19]. In a large normative first-trimester cohort, Altmann et al. identified one case of isolated GE dysplasia in 402 fetuses (0.25%), which was later confirmed postnatally as cobblestone lissencephaly associated with a 16p13.11 deletion [24].

Birnbaum et al. reported GE cavitation as the sole inclusion criterion, with bilateral cysts in 64% (16/25) and unilateral cysts in 36% (9/25) of cases [26]. Notably, in four cases the initial GE cyst subsequently regressed and transformed into GE enlargement on follow-up imaging, suggesting that cavitation and enlargement may represent a pathological continuum rather than distinct entities. Chen et al. reported persistent GE or GE cavitation as a combined neurosonographic sign (sign I), without separating the two phenotypes; genetic MCD was identified in 66.7–73.7% of fetuses with this sign who underwent complete genetic testing [30].

### 3.7. Clinical Associations: CNS Malformations and Genetic Findings

In MRI-based cohorts, GE abnormalities were associated with a range of cortical malformations. Scarabello et al. reported lissencephaly in 21%, heterotopia in 18%, schizencephaly in 25%, and polymicrogyria in 22% of cases [19]. Goergen et al. found head size below the 5th centile in 68% (15/22) of fetuses with GE abnormalities, and coexisting anomalies included schizencephaly and heterotopia [18]. Righini et al. reported coexisting ventriculomegaly, corpus callosum agenesis, and cerebellar hypoplasia [17]. In a neurosonography cohort of 50 fetuses with MCD, Contro et al. reported polymicrogyria in 32%, lissencephaly in 24%, and corpus callosum agenesis in 18% [21]. Across combined cohorts, 59–100% of fetuses with GE abnormalities had coexisting MCD or genetic diagnoses [18,19].

Genetic testing (chromosomal microarray and/or exome sequencing) was performed in 65–82% of cases with abnormal GE findings, with a diagnostic yield of 40–71% [18,19,26,30]. Identified variants included KCNQ1OT1-AS1 duplication and 22q11 deletion [18], and a 16p13.11 deletion in the isolated GE dysplasia case reported by Altmann et al. [24]. Delivery in a tertiary care center was reported in 85–100% of cases across cohorts with GE abnormalities [18,19].

The two studies added in the updated search substantially expand the data on clinical associations. Birnbaum et al. reported additional brain abnormalities in 81.8% (18/22) of assessable fetuses, most commonly corpus callosum malformations (92.3%), periventricular hyperechogenicity (71.4%), and thin hemispheric parenchyma (64.3%) [26]. Extracranial anomalies were present in 68% (17/25) of cases, including skeletal abnormalities (35.3%), cardiovascular defects (29.4%), and fetal growth restriction (35.3%). Genetic testing was performed in 73.9% of families, with a diagnostic yield of 70.6% (12/17); the predominant genetic mechanism was mitochondrial dysfunction, involving five different nuclear genes (POLG, EARS2, COQ6, NDUFAF5, PDHA1), followed by tubulinopathies, channelopathy, and RASopathy. This diagnostic yield of 70.6% substantially exceeds the 40–59% reported in earlier cohorts, likely reflecting the combined use of chromosomal microarray and exome sequencing, including whole-genome sequencing in selected cases.

Chen et al. reported that among 95 fetuses with at least one of 10 predefined NSG signs of MCD, persistent GE or GE cavitation (sign I) was associated with a genetic MCD diagnosis in 66.7–73.7% of cases with complete genetic testing (CMA and exome sequencing) [30]. The most common coexisting anomalies among all 31 genetically confirmed MCD cases were ventriculomegaly (*n* = 16), posterior fossa anomalies (*n* = 9), and callosal dysgenesis (*n* = 7). These findings are consistent with the existing evidence that GE abnormalities rarely occur in isolation and are frequently part of a broader pattern of cortical maldevelopment with an underlying genetic etiology.

### 3.8. Diagnostic Pitfalls: Confusion with Choroid Plexus Cysts

GE cavitation may be misclassified as a choroid plexus cyst (CPC) on routine ultrasound. Brusilov et al. described three fetuses at 15–20 weeks in whom GE cavitation was initially misdiagnosed as CPC before dedicated neurosonography was performed [28]. Paladini et al. reassessed seven fetuses referred for large CPC at 21–35 weeks using neurosonography and confirmed GE cavitation in 4/7 cases (57%); cortical gyration abnormalities were found in 57% of those cases [27]. The key anatomical distinguishing feature is the anterior location of GE cavitation relative to the choroid plexus [27,28]. Prefumo et al. reported prenatal ultrasound detection of GE cavitation in two fetuses (16 and 20 weeks), with findings comparable to those on MRI [31].

## 4. Discussion

This systematic review supports the clinical concept that the fetal ganglionic eminence can be assessed prenatally as part of a structured neurosonographic evaluation for malformations of cortical development [32,33]. Across the fetal MRI literature, GE anomalies tend to divide into two main patterns: cavitations and increased GE volume. These patterns are frequently accompanied by other developmental brain anomalies rather than representing an isolated finding [18]. This is clinically relevant because ultrasound assessment of cortical development is often challenging, relies on indirect and sometimes subjective signs, and may lead to false-positive concerns if not integrated into a standardized approach [34]. In this context, dedicated neurosonography and fetal MRI should be considered complementary modalities that together improve detection, phenotyping, and counseling in suspected cortical and migration disorders [35].

Fetal brain appearance changes throughout gestation, so any GE-focused ultrasound approach must be interpreted in a gestational-age-specific context. Early targeted neurosonography may be technically advantageous because thin skull bones allow evaluation from multiple angles, improving visualization of deep structures compared with later gestation [36]. At the same time, some cortical malformations only become visible in the third trimester when gyration and sulcation are more advanced [37]. This timing issue mainly applies to indirect markers, such as abnormal fissure morphology or delayed folding, which are gestational-age dependent and may be interpreted subjectively [32]. However, ultrasound remains relatively insensitive to some transient compartments and small substructures of the developing fetal brain, which explains why GE-related abnormalities may be missed without dedicated acquisition and expertise.

Routine fetal screening in most settings is performed using a transabdominal approach, with limited opportunity for systematic assessment of deep intracranial structures. In contrast, transvaginal neurosonography should be considered a targeted, second-line modality within a dedicated neurosonography workflow, used when high-resolution imaging is clinically indicated and transvaginal access is feasible. The ISUOG 2021 practice guidelines recommend the transvaginal approach as the preferred method for high-resolution targeted neurosonographic examination [36]. Patient acceptability for transvaginal ultrasound in early pregnancy appears generally high when preceded by proper counseling [38,39].

A consistent observation from MRI studies is that most GE anomalies fall into two broad categories: cavitations and increased volume, usually accompanied by other structural brain anomalies [18,19]. In a multicenter MRI cohort, bilateral GE cavitations were the most prevalent abnormality and were frequently associated with severe cortical phenotypes within the spectrum of microlissencephaly and cerebellar anomalies [18]. These findings support a practical approach in prenatal counseling: when a GE abnormality is detected, it should prompt a systematic search for coexisting anomalies and referral for multidisciplinary evaluation, because GE abnormalities are rarely isolated and are usually part of broader malformations of the fetal brain [18,19,36].

A key clinical challenge is that, on routine ultrasound, GE cavitations may mimic apparently benign choroid plexus cysts, which can delay recognition of a potentially higher-risk developmental phenotype [27,28]. This diagnostic overlap underscores the importance of lesion location relative to the choroid plexus and the need for dedicated neurosonography in atypical cases.

Dedicated transvaginal neurosonography with 3D acquisition and parasagittal planes improves the chance of identifying subtle GE morphology, including cavitation and enlargement. In several series using targeted protocols, ultrasound findings were sufficient to detect GE abnormalities and guide further evaluation [22,25,31]. Nevertheless, MRI adds considerable value when ultrasound raises suspicion or cannot provide confident characterization, offering superior contrast and detailed phenotyping of associated anomalies [17,18,19]. Current guidelines therefore position neurosonography as the first targeted step, with MRI reserved for confirmation and comprehensive assessment [36]. A direct comparison of the advantages and disadvantages of both modalities is presented in Table 4.

Reproducibility of GE measurements was formally evaluated in only two prospective studies, with reported intraclass correlation coefficients (ICC) ranging from 0.64 to 0.90 for inter- and intraobserver variability. According to commonly accepted benchmarks, ICC values between 0.75 and 0.90 indicate good reliability, while values of 0.60–0.74 reflect moderate reliability. The observed range therefore suggests that GE biometry can achieve acceptable to good reproducibility when performed by experienced operators using standardized transvaginal neurosonography protocols. However, the lower end of this range (0.64) highlights that measurement consistency may still be suboptimal in less experienced hands or with non-standardized techniques. This moderate-to-good reproducibility supports the feasibility of incorporating GE assessment into dedicated neurosonographic examinations, but also underscores the need for proper training and protocol standardization before wider clinical implementation.

The two recently included studies expand the available evidence, although their findings should be interpreted in light of the selected referral populations involved. Birnbaum et al. described a retrospective multicenter case series of fetuses with GE cysts. In these cases, GE abnormalities were detected early by dedicated transvaginal neurosonography, and genetic testing revealed pathogenic variants in 12 of 17 examined fetuses; mitochondrial disorders dominated among the diagnoses [26]. Chen et al., examining a broader group of fetuses referred for suspected malformations of cortical development, observed that persistent GE or GE cavitation was linked to a genetic diagnosis in a substantial share of fully tested cases [30].

These results support the use of detailed imaging and etiological assessment when an abnormal GE appearance is found. However, they do not provide phenotype-specific diagnostic risk estimates, because both investigations enrolled high-risk cohorts and applied different inclusion criteria.

Birnbaum et al. also drew attention to the temporal evolution of GE abnormalities: lesions initially labeled as isolated unilateral GE cysts sometimes regressed and later evolved into GE enlargement, with occasional complete resolution on subsequent imaging [26]. This observation requires cautious interpretation. Physiological involution of the GE occurs between 20 and 28 weeks of gestation, and the clinical meaning of apparent sonographic normalization after an earlier abnormal finding remains unclear. Consequently, a GE abnormality identified early in gestation should trigger a systematic search for associated anomalies. Decisions concerning fetal MRI, genetic testing, and follow-up imaging should be individualized within a multidisciplinary evaluation.

In other words, the transient visibility of the GE can be misleading. Even if cavitation or enlargement resolves on later scans, this does not reverse the disruption that has already occurred in cortical architecture. This concept is supported by Righini et al., who reported that GE anomalies were not visible on routine ultrasound in any of their eight cases, despite clear associated brain malformations on MRI [17]. Similarly, Birnbaum et al. observed that early GE cysts frequently regressed or changed appearance during pregnancy, yet adverse neurodevelopmental outcomes remained common among survivors [26]. For clinical practice, this means that a normal ultrasound in the late second or third trimester cannot reliably exclude a prior GE insult. Once a GE abnormality has been detected at any gestational age, recommendations for fetal MRI, genetic testing, and structured neurodevelopmental follow-up should continue, regardless of subsequent sonographic improvement.

The biological meaning of GE abnormalities is consistent with the role of this transient subpallial compartment in the generation, differentiation, and migration of neuronal populations during early forebrain development. Disruption of these processes may plausibly coexist with abnormalities of cortical organization, including altered gyration and neuronal migration disorders. However, an abnormal prenatal GE appearance should be regarded as a non-specific imaging marker rather than evidence of a defined pathogenic mechanism. The studies included in this review were small, heterogeneous, and largely based on selected referral populations; consequently, they cannot determine whether individual GE phenotypes directly predict a particular malformation, genetic diagnosis, or neurodevelopmental outcome. In clinical practice, an abnormal GE should therefore prompt a targeted search for associated cerebral findings and individualized consideration of complementary imaging and etiological evaluation.

Prenatally detected GE abnormalities are rarely isolated and usually occur within a broader developmental context that includes other cortical malformations and underlying genetic etiologies. Long-term prognosis after prenatally detected malformations of cortical development is highly heterogeneous, ranging from normal outcomes to severe neurological deficits [32,35,40]. A critical limitation for counseling is that none of the included studies provided structured long-term neurodevelopmental follow-up for children with prenatally identified GE abnormalities [18,19,21,24]. As a result, the true prognostic value of GE assessment remains uncertain. This mirrors the broader field, where evidence is restricted by selective follow-up and high rates of pregnancy termination [32,40].

The relationship between genetic variants and the resulting brain malformations involving the ganglionic eminence is complex and often non-linear, which has important implications for prenatal counseling. In general, GE abnormalities associated with macrocephaly are frequently linked to mTORopathies, particularly mutations in PI3K, mTOR, and TSC1/TSC2 genes, leading to megalencephaly, hemimegalencephaly, or polymicrogyria with cortical overgrowth [41,42]. In contrast, GE abnormalities accompanied by microcephaly are strongly associated with tubulinopathies (e.g., TUBA1A, TUBB2B, TUBB3), which disrupt microtubule-dependent neuronal migration and commonly result in lissencephaly, pachygyria, or corpus callosum dysgenesis [41,42,43].

Specific genes show characteristic patterns: LIS1 mutations typically cause posterior-predominant lissencephaly, while DCX mutations lead to anterior lissencephaly in males and subcortical band heterotopia in females [44]. ARX mutations are particularly relevant because this gene regulates the tangential migration of GABAergic interneurons from the GE; its variants can cause a broad spectrum ranging from X-linked lissencephaly with abnormal genitalia to epileptic encephalopathy [40,41,44]. Periventricular heterotopia is most often caused by FLNA mutations, while somatic mosaic mutations in the PI3K-AKT-mTOR pathway can produce focal cortical dysplasia or hemimegalencephaly that may be missed on standard blood-based genetic testing [41,45,46].

Additionally, metabolic and mitochondrial disorders are increasingly recognized; GE cavitation has been linked to PDHA1 and ECHS1 deficiencies [18,26]. The recent study by Birnbaum et al. further highlighted mitochondrial dysfunction as a recurrent finding in fetuses with GE cysts [26]. Collectively, these findings emphasize that a normal chromosomal microarray does not rule out a clinically significant genetic diagnosis. Therefore, trio exome sequencing (with a diagnostic yield of approximately 27% in complex brain anomalies) should be systematically offered when GE abnormalities are detected prenatally, ideally guided by the specific imaging phenotype [47].

The clinical value of prenatal GE assessment lies in its potential as an early marker of severe neurodevelopmental disorders. Identification of GE abnormalities prompted fetal MRI in 91–100% of cases, genetic testing in 65–82% (with a diagnostic yield of 40–71%), and planned delivery in a tertiary center in 85–100% of cases. The relationship between different GE phenotypes and specific types of MCD is summarized in Table 5. Cavitation appears to be more frequently associated with lissencephaly and heterotopia, whereas enlargement is predominantly linked to polymicrogyria and schizencephaly. The ultrasonographic characteristics of normal GE, GE cavitation, GE enlargement, and choroid plexus cysts, including gestational age at detection, anatomical location, natural course, and associated anomalies are summarized in Table 6.

The high genetic diagnostic yield associated with GE abnormalities (40–71% in the included studies) raises the question of whether current clinical guidelines adequately reflect the importance of this finding, even when the GE anomaly appears isolated on ultrasound without other visible malformations. Current ISUOG guidelines recommend fetal MRI and genetic testing when GE abnormalities are detected, but do not clearly define the threshold for genetic evaluation in apparently isolated cases [36].

Data from this review and from Birnbaum et al. suggest that this threshold should be low [44]. In the multicenter study by Goergen et al., although extracranial findings (such as macrocephaly, cardiac rhabdomyoma, or polydactyly) helped narrow the genetic differential, a substantial proportion of fetuses with GE abnormalities still had pathogenic variants even without obvious associated anomalies [18]. Moreover, as discussed earlier, an apparently isolated GE abnormality on ultrasound may already be accompanied by subtle cortical changes that are not yet detectable by sonography. A normal ultrasound later in pregnancy therefore cannot reliably exclude a prior developmental insult.

The recently published retrospective study by Chen et al. provides additional quantitative support for a proactive genetic testing approach [30]. For 95 fetuses referred for neurosonography on suspicion of MCD, the authors analyzed ten discrete ultrasound signs and their association with pathogenic germline variants detected by chromosomal microarray (CMA) combined with exome sequencing. Persistent GE or GE cavitation (sign I) was associated with a genetic diagnosis in 66.7–73.7% of cases in which complete genetic testing was performed, placing it among the mid-to-high probability signs alongside irregular ventricular borders. Critically, the study demonstrated that the vast majority of ultrasound signs for MCD, including GE-related signs, are not gene-specific: the same sonographic pattern may result from mutations in entirely different genes and pathways. The authors therefore conclude that CMA combined with next-generation sequencing is the preferred diagnostic strategy once any of these signs is identified, rather than targeted single-gene testing. This finding directly reinforces the recommendation that detection of GE abnormality on neurosonography, even without a distinctive morphological pattern, should prompt comprehensive genetic evaluation rather than sequential or targeted testing.

Taken together, these findings support a proactive approach. We propose that any GE abnormality, including apparently isolated cavitation or enlargement, should prompt referral for fetal MRI and comprehensive genetic testing, at minimum chromosomal microarray, with trio exome sequencing offered if the microarray is normal. This recommendation is consistent with the higher diagnostic yield of exome sequencing (approximately 27%) compared with microarray alone (approximately 3%) in complex fetal brain anomalies [47]. Ultimately, while patient preferences and appropriate counseling must always be considered, clinicians should regard GE abnormalities as a strong indication for thorough etiologic investigation, even when they appear isolated on ultrasound.

This systematic review has several limitations. The most important is the inability to perform a quantitative meta-analysis due to the small number of eligible studies (*n* = 13) and substantial methodological heterogeneity. Included studies differed markedly in gestational age windows, imaging protocols, population characteristics, and definitions of GE abnormalities. Data on phenotypic spectrum were derived predominantly from case reports and small series, which are susceptible to selection bias. Long-term neurodevelopmental follow-up was largely absent. Finally, the literature search was restricted to three major databases and English-language publications.

Future work should prioritize standardization and validation of neurosonographic protocols that incorporate ganglionic eminence assessment. Prospective studies with long-term neurodevelopmental follow-up using standardized instruments are urgently needed. Imaging pathways should also integrate genetic evaluation, particularly when GE abnormalities are identified, as imaging alone is often insufficient to determine etiology and recurrence risk [40,48].

## 5. Conclusions

The available evidence indicates that transvaginal neurosonography can provide feasible and reproducible visualization of the fetal ganglionic eminence, particularly when dedicated three-dimensional protocols are used in the early and mid-second trimester. Abnormal GE appearances, including cavitation and enlargement, have been reported in association with malformations of cortical development and other central nervous system anomalies. However, the current evidence base is limited by small, heterogeneous, and predominantly high-risk cohorts, with scarce long-term neurodevelopmental follow-up. GE assessment may therefore be a useful component of targeted fetal neurosonography and may support referral for detailed imaging and etiological evaluation when an abnormality is identified. Its diagnostic and prognostic value, and its role in routine clinical assessment, require confirmation in prospective multicenter studies using standardized imaging protocols.

## Figures and Tables

**Table 1:** Selected studies characteristics.

No.	First Author (Year)	Study Design	GA (Weeks)	Fetuses (*n*)	Imaging Protocol	Main Findings
1	Righini (2016) [17]	Retrospective cohort	19–29	8	US + MRI	GE anomalies seen only on MRI
2	Goergen (2021) [18]	Prospective multicenter	21–33	22	US + MRI	Genetic diagnosis in 59%
3	Scarabello (2021) [19]	Retrospective multicenter	17–33	60	US + MRI	Cavitation (*n* = 34), enlargement (*n* = 26)
4	Boitor-Borza (2015a) [22]	Retrospective	9–13	18	3D TVUS (HDlive)	GE visualized in 83%
5	Boitor-Borza (2015b) [23]	Prospective pilot	7–10	4	3D/4D TVUS	GE visualized in 100%
6	Brusilov (2021) [28]	Case report	15–20	3	TV neurosonography	GE cavitation misdiagnosed as CPC
7	Prefumo (2019) [31]	Case report	16–20	2	Ultrasound	GE cavitation detected
8	Paladini (2021) [27]	Case series	21–35	7	TV neurosonography	GE cavitation in 4/7 cases
9	Altmann (2023) [24]	Prospective cross-sectional	11+3–13+6	402	3D TVUS	GE visualized in 71%
10	Contro (2023) [21]	Prospective multicenter	19–22	160	3D neurosonography	GE visualized in 90%
11	Birnbaum (2023) [25]	Retrospective	14–18	84	3D TVUS	GE visualized in 100%
12	Chen (2025) [30]	Retrospective cohort	19–38	95	TV and TA neurosonography	GE sign predicts genetic MCD
13	Birnbaum (2026) [26]	Retrospective multicenter case series	11–22	25	3D TV neurosonography	Early GE cysts; genetic associations

**Table 2:** Outcome of the included studies.

No.	First Author (Year)	Feasibility of GE Visualization (*n*/%)	Reproducibility (Intra/Interobserver ICC)	Cavitation (*n*)	Enlargement (*n*)	Persistence (*n*)
1	Righini (2016) [17]	N/A (US did not detect GE anomalies)	N/A	0	0	0
2	Goergen (2021) [18]	N/A (US extracranial focus)	N/A	0	15	7
3	Scarabello (2021) [19]	N/A (GE associated with CNS anomalies)	N/A	34	26	0
4	Boitor-Borza (2015)—Med Ultrason [22]	15/18 (83%)	N/A	0	0	0
5	Boitor-Borza (2015)—Clujul Med [23]	4/4 (100%)	N/A	0	0	0
6	Brusilov (2021) [28]	3/3 (100%)	N/A	3	0	0
7	Prefumo (2019) [31]	2/2 (100%)	N/A	2	0	0
8	Paladini (2021) [27]	7/7 (100%)	N/A	4	0	0
9	Altmann (2023) [24]	285/402 (71%)	Intra: almost perfect; Inter: ICC 0.64–0.90	0	0	0
10	Contro (2023) [21]	144/160 (90%)	Intra 0.80–0.90; Inter 0.64–0.90	4	12	0
11	Birnbaum (2023) [25]	84/84 (100%)	N/A	0	0	0
12	Chen (2025) [30]	N/A (Not reported as primary outcome)	N/A	Reported as sign I (persistent GE or GE cavitation)—exact n not specified separately *		0
13	Birnbaum (2026) [26]	25/25 (100%)	N/A	25	4	0

* Chen et al. (2025) [30] defined persistent ganglionic eminence and GE cavitation as a single neurosonographic sign (sign I); the two phenotypes were not reported separately.

**Table 3:** The feasibility pattern across gestation.

Gestational Age Window	Visualization Rate	Study [Ref]
7–10 weeks	100% (4/4)	Boitor-Borza [22]
9–13 weeks	83% (15/18)	Boitor-Borza [23]
11+3 to 13+6 weeks	71% (285/402); measurement ≥ 1 dimension 57% (229/402)	Altmann [24]
14–18 weeks	100% (84/84)	Birnbaum [25]
19–22 weeks	90% (144/160)	Contro [21]

**Table 4:** Comparison of dedicated transvaginal neurosonography versus fetal MRI for ganglionic eminence assessment.

Aspect	Dedicated Transvaginal Neurosonography (TVS)	Fetal MRI	TVS + MRI (Combined Approach)
Availability	High (most fetal medicine centers)	Moderate (limited access)	Optimal
Gestational age suitability	Excellent from 9–22 weeks	Good from 18 weeks onward	Best coverage across gestation
Cost	Low	High	Moderate
Real-time imaging	Yes	No	Yes (TVS)
Spatial resolution	Very high (near-field)	High (whole brain)	Highest
Patient comfort/acceptability	Good (after counseling)	Moderate (claustrophobia, noise)	Good
Detection of subtle GE changes	Excellent	Very good	Excellent
Detailed phenotyping of MCD	Moderate	Excellent	Excellent
Need for sedation	No	Sometimes (late gestation)	Rarely
Diagnostic speed	Immediate	Delayed (appointment required)	Rapid triage + confirmation

**Table 5:** Coexisting malformations of cortical development reported in included cohorts with ganglionic eminence abnormalities.

GE Abnormality Type	Most Commonly Associated MCD	Frequency Reported in the Relevant Included Cohort	Other Common Findings	References
Cavitation	Lissencephaly, Heterotopia	18–21%	Delayed gyration, cerebellar anomalies	[19]
Enlargement	Polymicrogyria, Schizencephaly	22–32%	Macrocephaly, corpus callosum anomalies	[19,21]
Persistence	Delayed cortical maturation	~32%	Opercularization defects	[18]

MCD = malformations of cortical development. Frequencies are derived from selected high-risk cohorts included in this review. Where the primary studies did not provide cross-tabulations between a specific GE phenotype and a specific MCD subtype, percentages describe cohort-level co-occurrence and should not be interpreted as phenotype-specific risk estimates.

**Table 6:** Ultrasonographic characteristics of normal GE, GE cavitation, GE enlargement, and choroid plexus cysts.

Feature	Normal GE	GE Cavitation	GE Enlargement	Choroid Plexus Cyst (CPC)
Gestational age at detection	7–22 weeks (peak 14–20)	15–28 weeks	14–32 weeks	16–24 weeks (usually resolves)
Anatomical location	Anterior/medial to CP, caudothalamic groove	Within GE tissue, anterior to CP	Bulging GE into lateral ventricle	Inside choroid plexus
Echogenicity	Homogeneous, iso- to mildly hyperechoic	Hypoechoic (cystic) areas	Increased size, hyperechoic	Hypoechoic, cystic
Natural course	Physiological involution by 28–32 weeks	Persistent or slowly resolving	May persist or regress	Usually resolves by 26–28 weeks
Associated anomalies	None	High (lissencephaly, heterotopia)	High (polymicrogyria, schizencephaly)	Low (usually isolated)
Clinical significance	Normal variant	Strong marker of MCD	Strong marker of MCD	Generally benign

## Data Availability

The datasets generated and analyzed during the current study are available from the corresponding author on reasonable request.

## Supplementary Materials

The following supporting information can be downloaded at: https://www.mdpi.com/article/10.3390/life16081362/s1, Table S1. Search strings for the literature databases; Table S2. Summary of risk of bias for cohort and case-control studies; Table S3. JBI Critical Appraisal Checklist for Case Reports; Figure S1. Risk of bias assessment of the included cohort and diagnostic accuracy studies (*n* = 9) using the QUADAS-2 tool; Figure S2. Risk of bias assessment of the included case reports and case series (*n* = 4) using the Joanna Briggs Institute (JBI) critical appraisal checklist.

## Abbreviations

3D	Three-dimensional
CNS	Central nervous system
CPC	Choroid plexus cyst
GE	Ganglionic eminence
ICC	Intraclass correlation coefficient
MCD	Malformations of cortical development
MRI	Magnetic resonance imaging
PRISMA	Preferred Reporting Items for Systematic Reviews and Meta-Analyses
PROSPERO	International Prospective Register of Systematic Reviews
