# Supplementary material for: The Value of Transvaginal Neurosonography in the Detection of Fetal Ganglionic Eminence Abnormalities
Source: Life (Basel). 2026 Aug 19;16(8):1362. doi: 10.3390/life16081362 (PMC13514536; doi:10.3390/life16081362)

Suplementary materials.

Table S1. Search strings for the literature databases.

Database	Search string
PubMed	("ganglionic eminence" [All Fields] or "fetal ganglionic eminence" [All Fields])AND ("diagnostic imaging"[MeSH Subheading] OR ("diagnostic"[All Fields] AND "imaging"[All Fields]) OR "diagnostic imaging"[All Fields] OR "ultrasound"[All Fields] OR "ultrasonography"[MeSH Terms] OR "ultrasonography"[All Fields] OR "ultrasonics"[MeSH Terms] OR "ultrasonics"[All Fields] OR "ultrasounds"[All Fields] OR "ultrasound s"[All Fields])
Web of Science	(ALL=(ganglionic eminence) OR ALL=(fetal ganglionic eminence)) AND (ALL=(diagnostic imaging) OR ALL=(ultrasound) OR ALL=(ultrasonography) OR ALL=(ultrasonics) OR ALL=(ultrasounds))
Scopus	(TITLE-ABS-KEY ("ganglionic eminence") OR TITLE-ABS-KEY ("fetal ganglionic eminence")) AND (TITLE-ABS-KEY ("diagnostic imaging") OR TITLE-ABS-KEY (ultrasound) OR TITLE-ABS-KEY (ultrasonography) OR TITLE-ABS-KEY (ultrasonics) OR TITLE-ABS-KEY (ultrasounds))

Table S2. Summary of risk of bias for cohort and case-control studies

**No.**	**First author (year)**	**Patient selection**	**Index test (US/neurosonography)**	**Reference standard (MRI/postnatal)**	**Flow and timing**	**Overall risk**	**Comments**
1	Righini (2016)	Low (consecutive cases)	High (general USG, no dedicated protocol)	Low (MRI 1.5T)	Low (same GA)	Moderate	Bias in index test; retrospective
2	Goergen (2021)	Low (prospective multicenter)	High (general USG, extracranial focus)	Low (MRI + genetic/postnatal)	Low (minimal discrepancy)	Moderate	Strong design, but US not GE-specific
3	Scarabello (2021)	High (retrospective selection)	High (general USG)	Low (MRI 1.5T)	High (variable GA)	High	High selection and timing bias

4	Boitor-Borza (2015) – Med Ultrason	Low (consecutive)	Low (3D TVUS + HDlive)	N/A (normative)	Low	Low	Low bias; normative retrospective
5	Boitor-Borza (2015) – Clujul Med	Low (prospective pilot)	Low (3D/4D TVUS)	N/A (pilot normative)	Low	Low	Low bias; small n but prospective
6	Altmann (2023)	Low (prospective cross-sectional)	Low (3D TVUS dedicated)	N/A (normative; 1 postnatal MRI)	Low	Low	Lowest bias; large n
7	Contro (2023)	Low (prospective multicenter)	Low (3D neurosonography protocol)	Low (MCD confirmation)	Low	Low	Lowest bias; multicenter, ICC assessed
8	Birnbaum (2023)	Low (retrospective analysis)	Low (3D TVUS)	N/A (normative)	Low	Low	Low bias; large n for retrospective
9	Chen (2025)	High (retrospective, single-center, selected on predefined US signs)	Low (10 predefined signs, dedicated NSG per ISUOG, experienced operators)	High (CMA+ES only in 40/95; misses somatic mutations; many declined/lost)	High (high attrition, differential verification, many TOP)	High	Retrospective single-center exploratory study on 10 US signs vs genetic MCD.

Table S3. JBI Critical Appraisal Checklist for Case Reports

No.	Question	Brusilov et al. (2021) (n=3)	Brusilov et al. (2021) – Comments	Prefumo et al. (2019) (n=2)	Prefumo et al. (2019) – Comments	Paladini et al. (2021) (n=7)	Paladini et al. (2021) – Comments	Birnbaum et al. (2026) (n=25)	Birnbaum et al. (2026) – Comments

1	Were patient's demographic characteristics clearly described?	Yes	Gestational age 15–20 weeks, relevant details (GA, parity) provided	Yes	Gestational age 16–20 weeks, maternal details implied	Yes	Gestational ages 21–35 weeks, consecutive series	Yes	Gestational age at diagnosis (range 11+3 – 22+4 weeks), cyst laterality, biometric data clearly reported for all 25 cases in Table 1
2	Was the patient's history clearly described and presented as a timeline?	Yes	Timeline from detection to follow-up (e.g., 15w detection, MRI)	Yes	Detection at 16/20w, follow-up timeline	Yes	Timeline from detection (21w) to follow-up (28–30w + postnatal)	Yes	Clear timeline: referral after screening → targeted TV neurosonography → genetic testing → pregnancy outcome and postnatal follow-up. Visible per case in Table 1

3	Was the current clinical condition of the patient on presentation clearly described?	Yes	Detailed US findings (cavitation anterior to CPC)	Yes	US findings (cavitation in GE)	Yes	Large CPC + GE cavitation in 57%	Yes	Very detailed sonographic features of GE cysts, classification of associated brain anomalies (midline, hemispheric, ventricular, sulcation, midbrain-hindbrain) and extracranial findings

4	Were diagnostic tests or assessment methods and the results clearly described?	Yes	Transvaginal neurosonography + MRI; results with images	Yes	US protocol (though not detailed); results with figures	Yes	Transvaginal neurosonography + MRI protocol; results detailed	Yes	Excellent. Clear diagnostic criteria for GE cyst, standardized targeted TV neurosonography, expert re-review of all images/volumes, structured anomaly classification, detailed genetic methods (CMA → ES/WGS)
5	Was the intervention(s) or treatment procedure(s) clearly described?	N/A	No intervention – diagnostic only	N/A	Diagnostic focus	N/A	Diagnostic series	N/A	Purely diagnostic study – no therapeutic interventions

6	Was the post-intervention clinical condition clearly described?	Yes	Postnatal outcomes (normal in 2, termination in 1)	Yes	Postnatal/ MRI confirmation	Yes	Follow-up MRI/postnatal outcomes (57% cortical anomalies)	Yes	Pregnancy outcomes clearly reported (TOP 64%, live birth 32%, 1 lost to FU). Postnatal data for survivors and autopsy findings where available
7	Were adverse events (harms) or unanticipated events identified and described?	Yes	Misdiagnosis as CPC highlighted as risk	Unclear	No explicit harms, but associated malformations noted	Yes	Misdiagnosis CPC (57%); associated MCD as harm	Yes	Clearly highlighted risk of misdiagnosis as choroid plexus cyst (CPC). High rate of associated severe anomalies and poor prognosis well documented

8	Does the case report provide takeaway lessons?	Yes	Emphasizes distinction from CPC and need for expert neurosonography	Yes	US can detect GE cavitation like MRI; emphasize location	Yes	Recommend neurosonography + MRI at 28–30w for large CPC	Yes	Strong clinical conclusions: need for expert early TV neurosonography, association with other anomalies and mitochondrial dysfunction, importance of comprehensive genetic testing (CMA + ES)

Figure S1. Risk of bias assessment of the included cohort and diagnostic accuracy studies (n=9) using the QUADAS-2 tool.

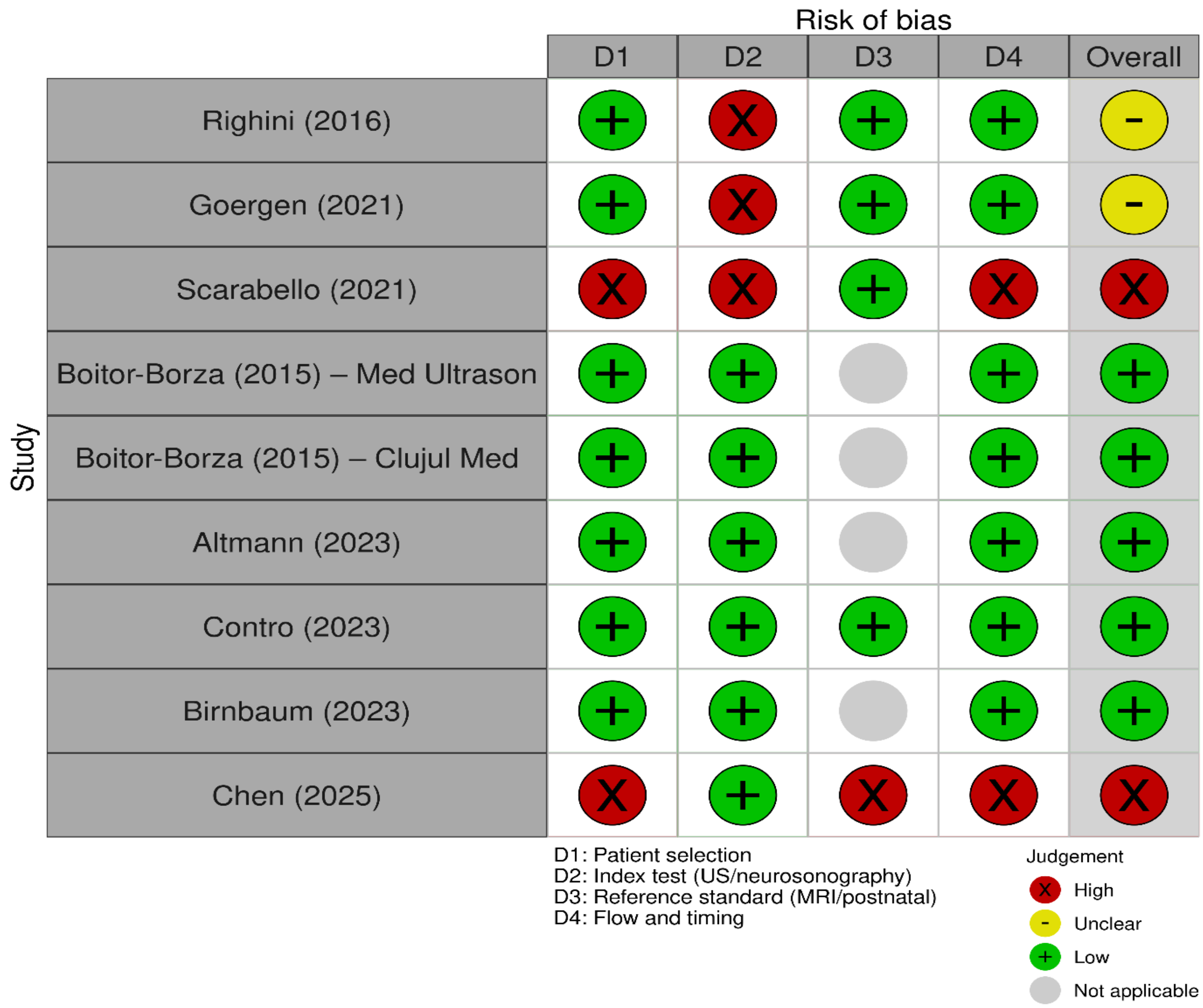

Figure S2. Risk of bias assessment of the included case reports and case series (n=4) using the Joanna Briggs Institute (JBI) critical appraisal checklist.

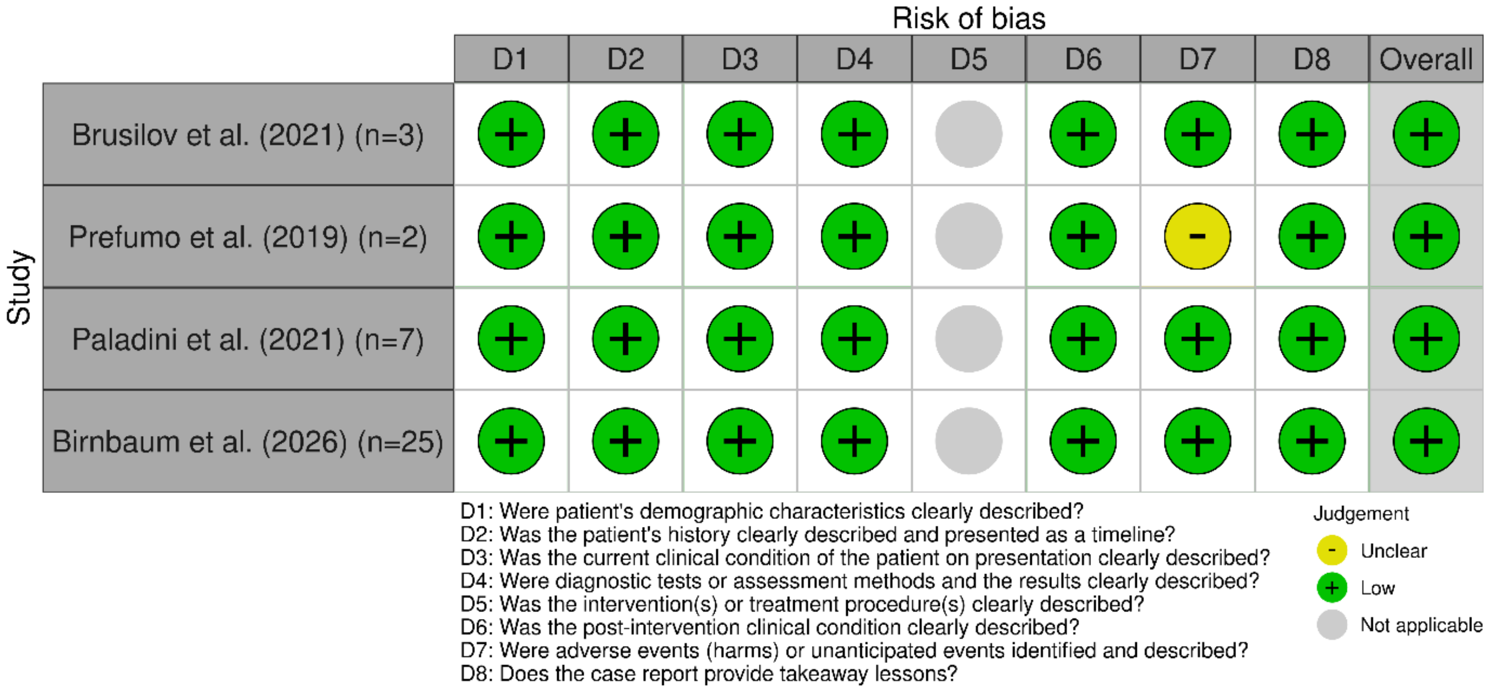

Supplement: Supplementary file 1 [file life-16-01362-s001.zip › life-4467789-supplementary.pdf]
